# Effect of non‐recommended doses versus recommended doses of direct oral anticoagulants in atrial fibrillation patients: A meta‐analysis

**DOI:** 10.1002/clc.23586

**Published:** 2021-03-07

**Authors:** Xuyang Liu, Manxiang Huang, Caisheng Ye, Xiujuan Xiao, Chengguang Yan

**Affiliations:** ^1^ Department of Cardiology Jinggangshan University Ji'an Jiangxi China

**Keywords:** anticoagulants, atrial fibrillation, off label, stroke prevention

## Abstract

**Background:**

Several observational studies have shown that the inappropriate dosing use of direct oral anticoagulants (DOACs) in atrial fibrillation (AF) that does not conform to recommendations is becoming a widespread phenomenon. Therefore, we performed a meta‐analysis and systematic review to assess the effect of non‐recommended doses versus recommended doses of DOACs on the effectiveness and safety outcomes among AF patients.

**Methods:**

The PubMed and Ovid databases were systematically searched to identify the relevant studies until December 2020. The effect estimates were hazard ratios (HRs) and 95% confidence intervals (CIs), which were pooled using a fixed‐effects model (I^2^ ≤ 50%) or a random‐effects model (I^2^ > 50%).

**Results:**

A total of 11 studies were included in this meta‐analysis. Compared with recommended dosing of DOACs, non‐recommended low dosing of DOACs was associated with increased risks of stroke or systemic embolism (SSE, HR = 1.29, 95% CI 1.12–1.49) and all‐cause death (HR = 1.37, 95% CI 1.15–1.62), but not the ischemic stroke, myocardial infarction, gastrointestinal bleeding, intracranial bleeding, and major bleeding. Compared with recommended dosing of DOACs, non‐recommended high dosing of DOACs was associated with increased risks of SSE (HR = 1.44, 95% CI 1.01–2.04), major bleeding (HR = 1.99, 95% CI 1.48–2.68), and all‐cause death(HR = 1.38, 95% CI 1.02–1.87).

**Conclusion:**

Compared with recommended dosing of DOACs, non‐recommended low dosing of DOACs was associated with increased risks of SSE and all‐cause death. Further study should confirm the findings of non‐recommended high dosing versus recommended dosing of DOACs.

## 1 INTRODUCTION

Atrial fibrillation (AF) is the most common cardiac arrhythmia, resulting in increased risks of stroke and its related mortality and disability.^1^ Anticoagulant drugs are effective drugs to prevent stroke risk in patients with AF.^2^ Previous randomized clinical trials (RCTs) and observational studies have consistently demonstrated that direct oral anticoagulants (DOACs, including dabigatran, rivaroxaban, apixaban, edoxaban) are at least non‐inferior in reducing the risks of stroke and bleeding compared with warfarin. Over the past decade, DOACs have gradually changed the landscape of anticoagulation treatment in AF because of the superior effectiveness and safety profiles of DOACs compared to warfarin. Dose adjustments for DOACs based on the European Medicine Agency, Food and Drugs Administration, and European Society of Cardiology guidelines have similarly recommended the use of (1) low dose of dabigatran (110 mg twice a day) if patients are aged ≥80 years, or have a creatinine clearance of 30–49 ml/min; (2) low dose of rivaroxaban (15 mg once a day) if patients have a creatinine clearance level of 30–49 ml/min; (3) low dose of apixaban (2.5 mg twice a day) if patients have any two of the following risk factors: age ≥ 80 years, a creatinine clearance level of ≥1.5 mg/dl, and bodyweight ≤60 kg; (4) low dose of edoxaban (30 mg once a day) if patients have a bodyweight ≤60 kg, creatinine clearance level of 30–50 ml/min, or concomitant P‐glycoprotein inhibitors.^3^, ^4^ Nevertheless, the use of reduced‐dose DOACs does not conform to the label‐ or guideline‐ recommendations in real‐world settings. The off label low or high dose of DOACs is regarded as non‐recommended low dose (underdosing) or non‐recommended high dose (overdosing), respectively. The reasons why the off label dose of DOACs is commonly used are still exploratory.

In these years, more and more researchers have explored the effect of non‐recommended low dose or non‐recommended high dose of DOACs among patients with AF.^5^, ^6^, ^7^, ^8^, ^9^, ^10^, ^11^, ^12^, ^13^, ^14^, ^15^ However, the findings of those observational studies are sometimes quite different; and thus, the effectiveness and safety profiles among non‐recommended doses of DOACs remain unclear, leaving physicians with difficulties in decision‐making regarding the choice of DOAC doses. Therefore, we conducted a meta‐analysis and systematic review to examine the effect of non‐recommended doses (underdosing or overdosing) versus recommended doses of DOACs on effectiveness and/or safety outcomes among AF patients.

## 2 METHODS

Since it was a meta‐analysis based on the published studies, ethical approval was not necessary. The data that support the findings of this study would be available from the corresponding author on the reasonable requests.

### 2.1 Literature search

The process of this meta‐analysis was conducted according to the guidance from the Cochrane Handbook for Systematic Reviews. The results of this meta‐analysis were presented according to the PRISMA (Preferred Reporting Items for Systematic Reviews and Meta‐analyses). The PubMed and Ovid electronic databases were systematically searched to identify the relevant studies that reporting the inappropriate DOAC dosing in AF patients. The retrieval periods were from January 2009 to December 2020 because the first publication of DOAC (dabigatran) in AF patients was reported in the year of 2009. As shown in Supplemental Table 1, the following index terms and their similar keywords were used in the electronic search: (1) “atrial fibrillation” OR “atrial flutter” AND (2) “direct oral anticoagulants” OR “non‐vitamin K antagonist oral anticoagulants” OR “dabigatran” OR “rivaroxaban” OR “apixaban” OR “edoxaban” AND (3) dose OR dosing OR overdosing OR underdosing. To reduce the omission of available and value studies, we scanned the reference lists of relevant meta‐analyses and reviews based on the DOAC dosing.^3^, ^16^, ^17^, ^18^, ^19^ We applied no linguistic restrictions in the search.

### 2.2 Inclusion and exclusion criteria

We included observational studies that reported the effect of non‐recommended doses (underdosing or overdosing) versus recommended doses of at least one DOAC (dabigatran, rivaroxaban, apixaban, or edoxaban) on effectiveness and/or safety outcomes among AF patients. The low or high dose of DOACs was defined as non‐recommended low dose (underdosing) or non‐recommended high dose (overdosing) respectively if they did not conform to the label‐ or guideline‐ recommendations. We applied the dosing criteria of DOACs in each included study. The effect estimates of this study were adjusted hazard ratios (HRs) and 95% confidence intervals (CIs). If several studies had overlapping data from the same data sources, we included the study with the longest follow‐up or highest sample size.

### 2.3 Effectiveness and safety outcomes

The primary effectiveness outcome was stroke or systemic embolism, whereas the primary safety outcome was major bleeding. Our secondary effectiveness outcomes included ischemic stroke, myocardial infarction, and all‐cause death, whereas the secondary safety outcomes were intracranial bleeding and gastrointestinal bleeding. We applied the original definitions of the effectiveness and safety outcomes in the included studies.

### 2.4 Study selection and data extraction

Two reviewers (Xuyang Liu and Manxiang Huang) independently screened the retrieved studies by reading the titles/abstracts to find out the potential studies, the full texts of which were subsequently reviewed for more information. Eligible studies were selected according to the inclusion criteria. Disagreements were resolved by consensus between the two reviewers, or discussion with a third reviewer (Chengguang Yan).

For each included study, two reviewers (Xuyang Liu and Manxiang Huang) independently extracted the data including study information (author, year of publication, design of the study, study period, data source, geographical characteristic), patient characteristics (sample size, age, sex), information of DOACs (type, dosage), follow‐up time, outcomes, and effect estimates.

### 2.5 Quality assessment

The Newcastle‐Ottawa Scale (NOS) items were used to assess the study quality of observational studies. The NOS tool involved three domains with a total of 9 points: the selection of cohorts (0–4 points), the comparability of cohorts (0–2 points), and the assessment of the outcome (0–3 points). One study was rated as a moderate‐to‐high quality if a NOS score of ≥6 points and as low quality if a NOS score of <6 points.^20^, ^21^

### 2.6 Statistical analysis

The statistical heterogeneity across the included studies was usually dealt with by the Cochrane Q test and I^2^ index. A *p* value of <.1 in the Cochrane Q test or an I^2^ value of >50% indicated significant heterogeneity between studies. The natural logarithms of the HRs and its corresponding standard errors were calculated. In the pooled analysis, the natural logarithms were pooled using a fixed‐effects model (I^2^ ≤ 50%) or a random‐effects model (I^2^ > 50%). For the primary outcomes, we used the inverse variance heterogeneity model or the quality effects model to re‐perform the meta‐analysis to test the robustness of our results. The subgroup analysis was performed based on the geographical characteristic (Asians vs. non‐Asians). Publication bias was evaluated by employing the funnel plots for visual inspection of asymmetry. We also used Egger's and Begg's tests for the reported outcomes to assess the potential publication bias statistically.

The analyses were performed using the Review Manager version 5.3 software (the Cochrane Collaboration 2014, Nordic Cochrane Centre Copenhagen, Denmark), the Stata software (version 15.0, Stata Corp LP, College Station, TX), and MetaXL (version 5.3). A value of *p* < .05 was considered statistically significant.

## 3 RESULTS

The flow chart of the literature retrieval of this meta‐analysis is shown in Supplementary Figure 1. A total of 11 studies were included in this meta‐analysis.^6^, ^7^, ^8^, ^11^, ^14^, ^15^, ^22^, ^23^, ^24^, ^25^, ^26^ The specific baseline characteristics of these included studies are shown in Table 1. All the included studies had a moderate‐to‐high quality with a NOS score of ≥6 points (Supplemental Table 2).

**TABLE 1:** Baseline characteristics of the included studies

References	Study design	Data source	Study duration	Number of patients	Age	Male ratio	Follow‐up period	Type of DOACs	Under dose or over dose of DOACs
Chan YH‐2020	Retrospective study	Chang Gung Memorial Hospital; China, Taiwan	01/2010–09/2018	11 275	74.2 years	58%	NA	Dabigatran	Under‐does:abigatran‐110 mg Over‐does: Dabigatran ‐150 mg
								Rivaroxaban	Under‐does: Rivaroxaban‐15 mg or 10 mg Over‐does: Rivaroxaban‐20 mg
								Apixaban	Under‐does: Apixaban‐2.5 mg Over‐does: Apixaban‐5 mg
								Edoxaban	Under‐does: Edoxaban‐30 mg Over‐does: Edoxaban‐60 mg
Cheng WH‐2019	Retrospective study	Taipei Veterans General Hospital; China, Taiwan	01/2012–12/2016	2214	75.7 years	64%	2.10 years	Rivaroxaban	Under‐does: Rivaroxaban‐10 mg
Lee SR‐2019	Retrospective study	Korean National Health Insurance Service database; Korea	01/2014–12/2016	10 392	NR	55%	1.4 years	Rivaroxaban	Under‐does: Rivaroxaban‐15 mg
Lee KH ‐2017	Retrospective study	Chonnam National University Hospital; Korea	01/2012–12/2013	844	NR	62.2%	1.0 year	Dabigatran	Under does: Dabigatran‐110 mg
Ikeda T‐2019	Prospective study	XAPASS; Japan	NR	6519	69.5 years	30.8%	0.84 years	Rivaroxaban	Under does: Rivaroxaban‐ 10 mg
Murata N‐2019	Prospective study	SAKURA AF; Japan	09/2013–12/2015	1658	71.7 years	71.5%	3.28 years	Dabigatran	Under does: Dabigatran‐110 mg
								Rivaroxaban	Under does: Rivaroxaban‐10 mg
								Apixaban	Under does: Apixaban‐2.5 mg
								Edoxaban	Under does: Edoxaban‐30 mg
Arbel R‐2019	Retrospective study	Clalit Health Services; Israel	01/2011–12/2017	8425	76.5 years	48.1%	1.92 years	Dabigatran	Unknown does
								Rivaroxaban	Unknown does
								Apixaban	Unknown does
Briasoulis A‐2020	Retrospective study	Medicare beneficiaries; United States	2010–2016	8035	NR	50.6%	1.08 years	Dabigatran	Under does: Dabigatran‐75 mg
								Rivaroxaban	Under does: Rivaroxaban‐15 mg
Yao X‐2017	Retrospective study	OptumLabs Data Warehouse; United States	10/2010–09/2015	3554	NR	NA	0.34 years	Dabigatran	Unknown does
								Rivaroxaban	Unknown does
								Apixaban	Unknown does
Steinberg BA‐2016	Retrospective study	ORBIT‐AF II; United States	2013–2016	5738	71.0 years	58.2%	0.99 years	Dabigatran	Under‐does: Dabigatran‐75 mg
								Rivaroxaban	Under‐does: Rivaroxaban‐ 15 mg
								Apixaban	Under‐does: Apixaban‐2.5 mg
								Edoxaban	Under‐does: Edoxaban‐ 30 mg
Camm AJ‐2020	Retrospective study	Global Anticoagulant Registry in the FIELD‐AF; Multicenter, 35 countries	2013–2016	10 426	74.0 years	55.9%	NA	Dabigatran	Under does: Dabigatran‐110 mg (EMA) or 75 mg (FDA)
								Rivaroxaban	Under‐does: Rivaroxaban‐15 mg
								Apixaban	Under‐does: Apixaban‐2.5 mg
								Edoxaban	Under‐does: Edoxaban‐30 mg

Abbreviations: DOACs, direct oral anticoagulants; NOS, Newcastle‐Ottawa Scale; NR, not reported.

### 3.1 Non‐recommended low dosing versus recommended dosing of DOACs

#### 3.1.1 Primary outcomes

**FIGURE 1:**
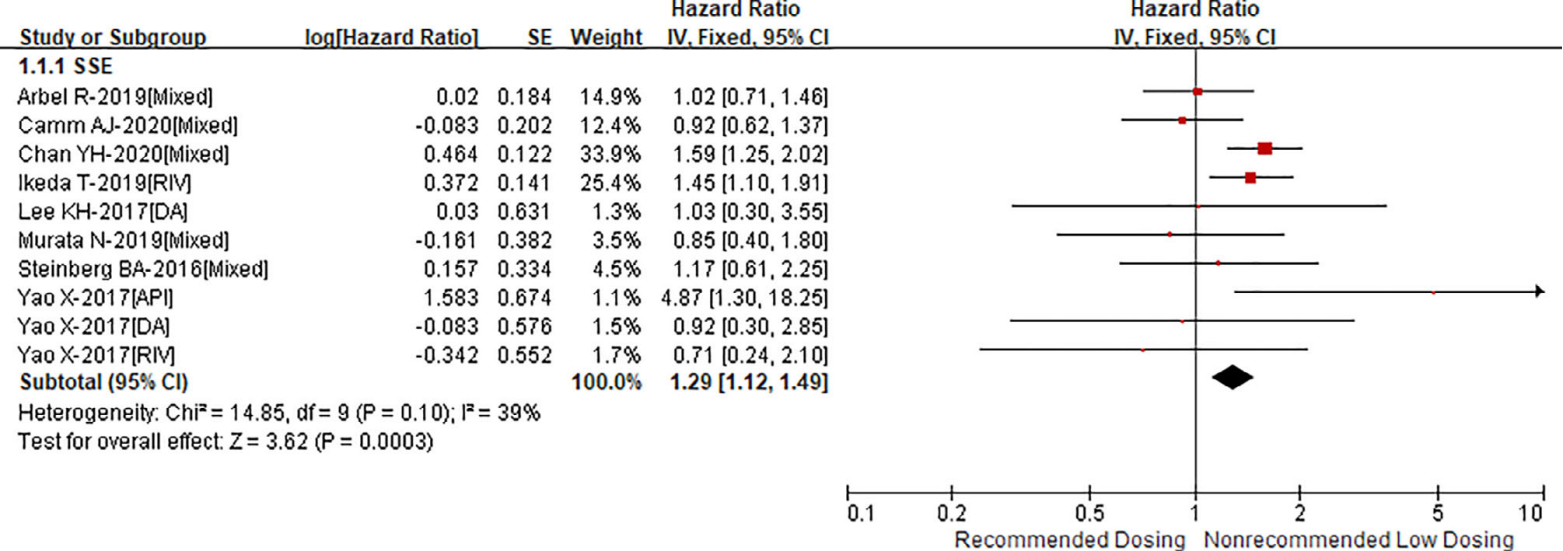
Comparing the outcome of stroke or systemic embolism between non‐recommended low dosing and recommended dosing of direct oral anticoagulants (DOACs)

**FIGURE 2:**
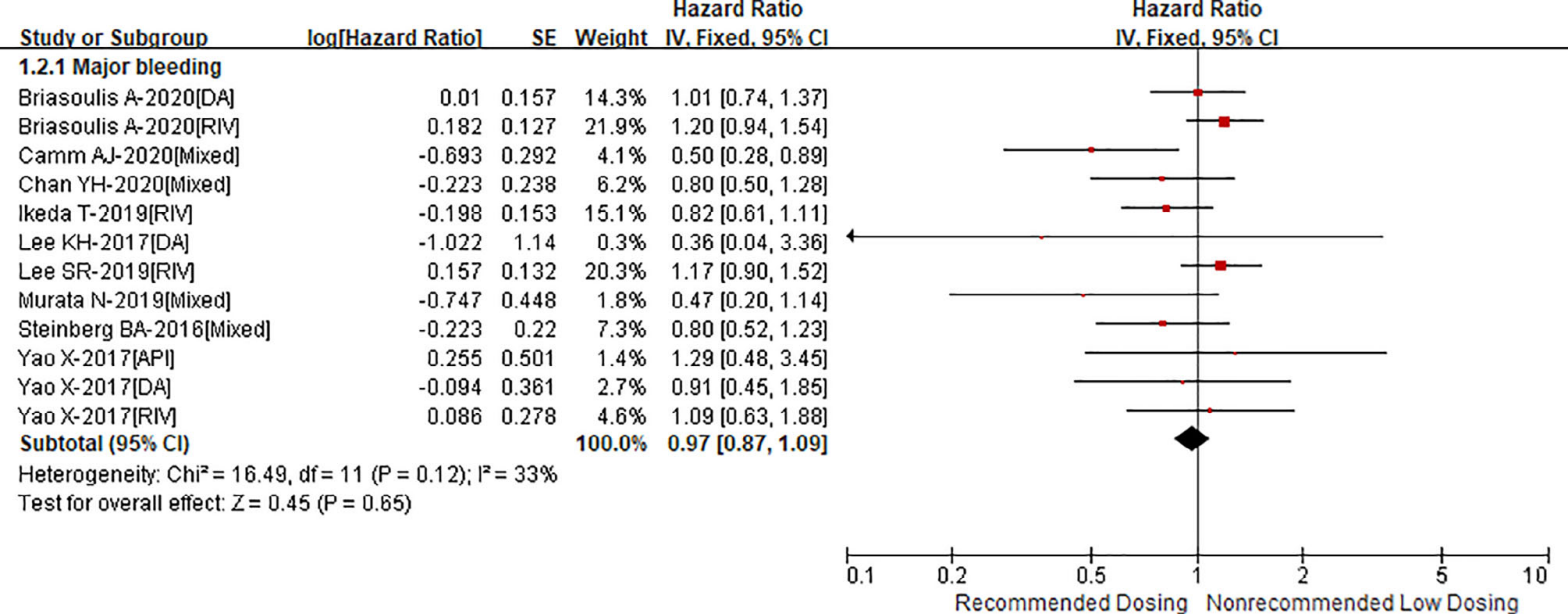
Comparing the outcome of major bleeding between non‐recommended low dosing and recommended dosing of direct oral anticoagulants (DOACs)

Compared with recommended dosing of DOACs, non‐recommended low dosing of DOACs was associated with an increased risk of stroke or systemic embolism (HR = 1.29, 95% CI 1.12–1.49; *p* = .0003; Figure 1) with an acceptable heterogeneity (I^2^ = 39%). However, the HR of stroke or systemic embolism was significant in Asians (HR = 1.37, 95% CI 1.17–1.60; *p* < .0001), but not in non‐Asians (HR = 1.22, 95% CI 0.77–1.96; *p* = .40) (P_interaction_ = 0.65; Supplementary Figure 2). As presented in Figure 2, there was no significant difference in the outcome of major bleeding (HR = 0.95, 95% CI 0.87–1.09; *p* = .65) between the two groups (I^2^ = 33%). The risk of major bleeding was similar in both Asian (HR = 0.94, 95% CI 0.79–1.12; *p* = .48) and non‐Asian (HR = 1.06, 95% CI 0.90–1.24; *p* = .49) patients (P_interaction_ = 0.32; Supplementary Figure 3). In addition, re‐analyses with an inverse variance heterogeneity or quality effects model suggested similar results as the above‐mentioned analysis with a fixed‐effects model.

#### 3.1.2 Secondary outcomes

As presented in Supplementary Figures 4 to 8, compared with recommended dosing of DOACs, non‐recommended low dosing of DOACs was associated with an increased risk of all‐cause death (HR = 1.37, 95% CI 1.15–1.62; *p* = .0004), but not the ischemic stroke (HR = 1.19, 95% CI 0.86–1.65; *p* = .29), and myocardial infarction (HR = 1.05, 95% CI 0.70–1.58; *p* = .82). For the secondary safety outcomes, no significant differences were found in the risks of gastrointestinal bleeding (HR = 1.14, 95% CI 0.96–1.37; *p* = .14) and intracranial bleeding (HR = 0.85, 95% CI 0.62–1.18; *p* = .33) between non‐recommended low dosing and recommended dosing of DOACs.

### 3.2 Non‐recommended high dosing versus recommended dosing of DOACs

#### 3.2.1 Primary outcomes

**FIGURE 3:**
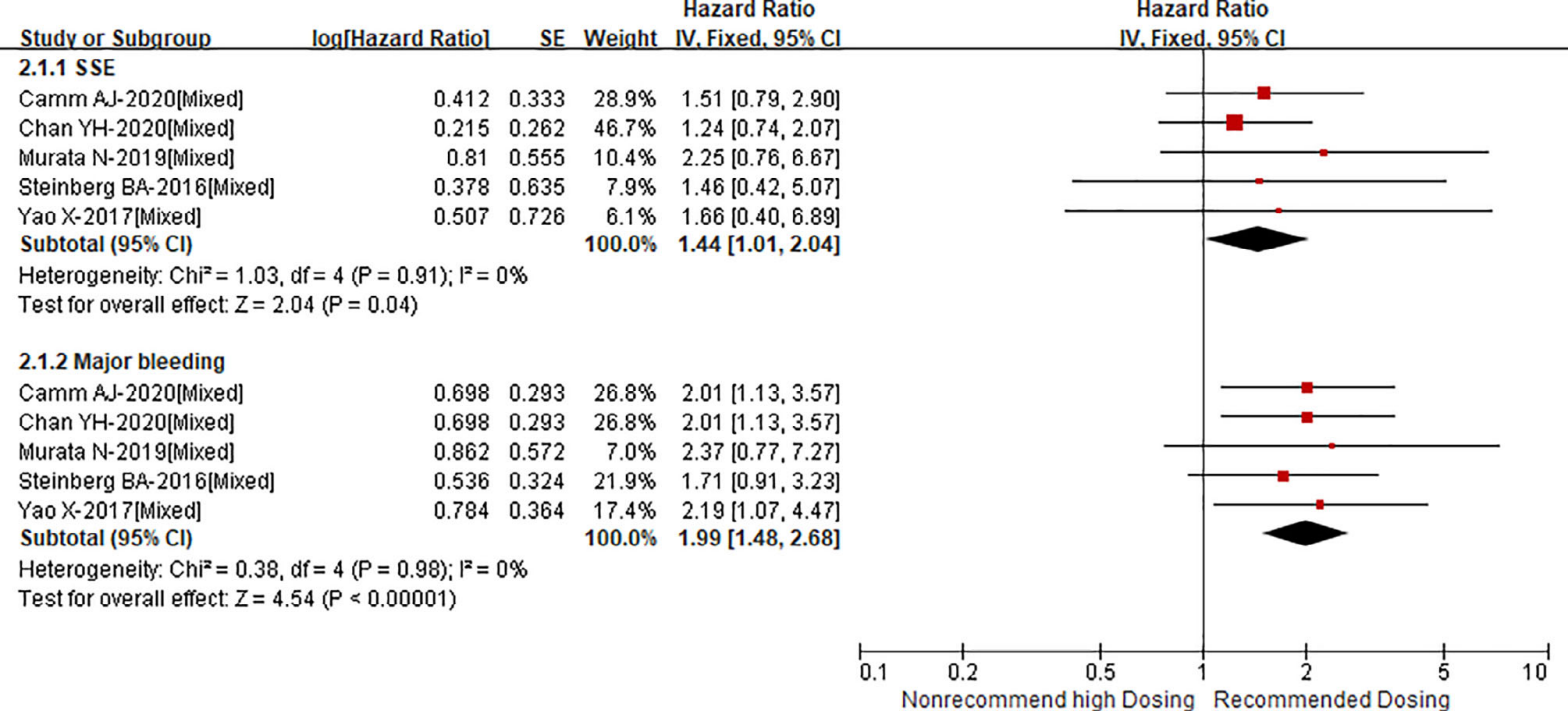
Comparing the outcome of stroke or systemic embolism and major bleeding between non‐recommended high dosing and recommended dosing of direct oral anticoagulants (DOACs)

As shown in Figure 3, compared with recommended dosing of DOACs, non‐recommended high dosing of DOACs was associated with increased risks of stroke or systemic embolism (HR = 1.44, 95% CI 1.01–2.04; *p* = .04) and major bleeding (HR = 1.99, 95% CI 1.48–2.68; *p* < .00001) with no heterogeneity (I^2^ = 0%). In addition, re‐analyses with an inverse variance heterogeneity or quality effects model suggested similar results. In the subgroup analysis, the risks of stroke or systemic embolism (Asian: RR = 1.38, 95% CI 0.87–2.20, and non‐Asian: RR = 1.54, 95% CI 0.60–3.94; P_interaction_ = 0.84) and major bleeding (Asian: RR = 2.08, 95% CI 1.25–3.47, and non‐Asian: RR = 1.91, 95% CI 1.19–3.06; P_interaction_ = 0.06) were similar in both Asians and non‐Asians (Supplementary Figures 9 to 10).

#### 3.2.2 Secondary outcomes

As shown in Supplementary Figure 11, compared with recommended dosing of DOACs, non‐recommended high dosing of DOACs was associated with an increased risk of all‐cause death (HR = 1.38, 95% CI 1.02–1.87; *p* = .04) with a low heterogeneity (I^2^ = 11%). The risks of ischemic stroke, myocardial infarction, intracranial bleeding, and gastrointestinal bleeding could not be assessed due to the limiting included studies.

### 3.3 Publication bias

There were no potential publication biases as assessed by inspecting the funnel plots (Supplementary Figures 12 to 13). For the group of non‐recommended low dosing versus recommended dosing, the Egger's (*p* = .402) and Begg's (*p* = .858) tests for the outcome of stroke or systemic embolism suggested no publication biases. For the outcome of major bleeding, the Begg's (*p* = .304) test suggested no publication bias, while Egger's test (*p* = .059) indicated certain publication bias. Nevertheless, the result from the trim‐and‐fill analysis showed no trimming performed, and the corresponding pooled data of major bleeding was not changed. For the group of non‐recommended high dosing versus recommended dosing, the Egger's and Begg's tests for the outcomes of stroke or systemic embolism and major bleeding indicated no significant publication biases (all *p* > .1).

## 4 DISCUSSION

In the present study, compared with recommended dosing of DOACs, non‐recommended low dosing of DOACs was associated with an increased risk of stroke or systemic embolism and all‐cause death, but not the ischemic stroke, myocardial infarction, gastrointestinal bleeding, intracranial bleeding, and major bleeding. Compared with recommended dosing of DOACs, non‐recommended high dosing of DOACs was associated with increased risks of stroke or systemic embolism, major bleeding, and all‐cause death. Based on our observed data, physicians should know the importance of appropriate NOAC dosing conforming to the label‐ or guideline‐ recommendations in clinical practice.

A systematic review by Bo et al.^19^ has previously found that non‐recommended doses of DOACs had no benefits of stroke prediction in AF patients because this inappropriate treatment might increase the risks of stroke and bleeding events. However, the study by Bo et al. was a descriptive analysis due to the limited quantitative data; and thus, the effectiveness and safety profiles among non‐recommended dose of DOACs remain unclear, leaving physicians with difficulties in decision‐making regarding the choice of DOAC doses. In these years, more and more researchers have explored the effect of non‐recommended low dose or non‐recommended high dose of DOACs in patients with AF, but their findings are quite different across the different data sources.

To our knowledge, we included more studies to quantitatively assess the effect of both underdosing and overdosing DOACs on adverse outcomes among AF patients. Our results suggested that compared with recommended dosing of DOACs, non‐recommended low dosing of DOACs could increase the risks of stroke or systemic embolism and all‐cause death. For the secondary outcomes, the risk for ischemic stroke was not increased in patients with non‐recommended low dosing of DOACs, while the risk for all‐cause death was higher among subjects with non recommended low dose, supporting the possibility that worse patients' condition resulted in physicians' prescribing a lower dose, and the poor condition was also associated with a worse prognosis. In addition, we also found that non‐recommended high dosing of DOACs could increase risks of stroke or systemic embolism and major bleeding. Herein, it was interesting to see the increased risk of stroke or systemic embolism in patients with non‐recommended high dosing of DOACs. In this part, we included five studies in the pooled analysis. As shown in Figure 3, all of the HRs were more than 1 across the included studies, although they were non‐significant. Therefore, the HR would be significant when increasing the sample size in the pooled analysis. The further studies should confirm these findings.

As we know, DOACs are different from each other, including differences in the pharmacological properties such as the extent of renal excretion and hepatic metabolism, in the specific criteria for dose adjustment, and the extent of the recommended dose reduction. Therefore, the effect of non‐recommended dose on clinical outcomes is expected to be different for different DOACs. However, since the number of included studies in these subgroups based on the type of DOACs was relatively small, the subgroup analysis based on the type of DOACs was not performed. Further clinical study could pay close attention to this issue.

Several studies have pointed out that there are some differences in oral anticoagulation treatment between Asian and non‐Asian patients with AF^27^, ^28^: (1) Asians have higher baseline risks of thromboembolism and bleeding than non‐Asians; and thus, more Asian patients would be ineligible for anticoagulation; (2) given the variations of genetic polymorphisms for warfarin metabolism in Asians, Asians are more sensitive to warfarin, which would result in an excessive bleeding risk; (3) Asians seemingly have a lower level of creatinine clearance, lower body weight, lesser use of gastric antacid drugs, and greater use of antiplatelets. These differences may affect the role of anticoagulation treatment in AF patients. Prior meta‐analyses based on evidence from RCTs and real‐world data have suggested that standard dose or low dose of DOACs are at least non‐inferior to warfarin in Asian patients with AF.^29^ In our current study, we performed the subgroup analysis based on the geographical characteristic (Asians vs. non‐Asians). For non‐recommended low dosing versus recommended dosing of DOACs, the HR of stroke or systemic embolism was significant in Asians but not in non‐Asians, while there was no significant difference in major bleeding between Asians and non‐Asians. For non‐recommended high dosing versus recommended dosing of DOACs, the risks of stroke or systemic embolism and major bleeding were similar in both Asian and non‐Asian patients.

### 4.1 Limitations

Of note, several limitations should be acknowledged in this meta‐analysis. First, since our study employed the real‐world data to conduct comparisons for efficacy and safety outcomes between non‐recommended doses (underdosing or overdosing) versus recommended doses of DOACs, residual confounders might exist and affect our findings. Second, the recommended dosing of DOACs was regarded as the reference in our study, which included both on‐label standard dose and a low dose of DOACs. It should be noted that some included studies only used on‐label standard‐dose of DOACs as the reference. Third, due to the limiting included studies, some secondary effectiveness and safety outcomes could not be assessed between non‐recommended high dosing versus recommended dosing of DOACs. Fourth, we only performed the subgroup analysis based on the geographical characteristic. However, the subgroup analyses based on the type of DOACs or other important parameters (e.g., age, sex, and sample size) were not performed due to the limiting data, which needs further studies.

## 5 CONCLUSIONS

Compared with recommended dosing of DOACs, non‐recommended low dosing of DOACs was associated with increased risks of stroke or systemic embolism and all‐cause death.. Further study should confirm the findings of non‐recommended high dosing versus recommended dosing of DOACs.

## CONFLICT OF INTEREST

All authors declare that they have no potential conflicts of interest that might be relevant to the contents of this review.

## AUTHOR CONTRIBUTIONS

Data curation: Xuyang Liu, Manxiang Huang. Formal analysis: Xuyang Liu, Manxiang Huang. Investigation: Xuyang Liu, Xiujuan Xiao. Methodology: Xuyang Liu, Caisheng Ye. Software: Xuyang Liu, Caisheng Ye. Supervision: Chengguang Yan. Validation: Chengguang Yan. Writing‐original draft:Xuyang Liu. Writing‐review and editing: Chengguang Yan.

## Supporting information


**Appendix S1.** Supporting Information.

## Data Availability

Availability of data and materials have been described in the manuscript. They are freely available to any scientist who wishes to use them without breaching participant confidentiality.
