# Supplementary material for: Effect of non‐recommended doses versus recommended doses of direct oral anticoagulants in atrial fibrillation patients: A meta‐analysis
Source: Clin Cardiol. 2021 Mar 7;44(4):472–80. doi: 10.1002/clc.23586 (PMC8027572; doi:10.1002/clc.23586)
Supplement: Supplementary file 1 — Appendix S1. Supporting Information. [file CLC-44-472-s001.docx]

**Supplementary Table 1. The search strategies in the PubMed database**

|  | **Queries** | **Items** |
| --- | --- | --- |
| **PubMed** | |  |
| #1 | atrial fibrillation OR atrial flutter | 89,068 |
| #2 | non-vitamin K antagonist oral anticoagulants OR NOACs OR direct oral anticoagulants OR DOACs OR new oral anticoagulants OR novel oral anticoagulants OR oral thrombin inhibitors OR oral factor xa inhibitors OR dabigatran OR rivaroxaban OR apixaban OR edoxaban | 19,284 |
| #3 | dose OR dosing OR overdosing OR underdosing OR off label | 1,425,660 |
| #4 | #1 and #2 and #3 and from 01/01/2009 to 10/19/2020 | 1,743 |

**Supplementary Table 2. Quality assessment of the included studies based on the Newcastle-Ottawa Scale (NOS) items**

| **Studies** | **Selection** | | | | **Comparability** | **Outcome** | | | **Total** |
| --- | --- | --- | --- | --- | --- | --- | --- | --- | --- |
|  | **Exposed**  **cohort** | **Non-exposed**  **cohort** | **Ascertainment**  **of exposure** | **Outcome**  **of interest** |  | **Assessment of**  **outcome** | **Length of**  **follow-up** | **Adequacy of**  **follow up** |  |
| Chan YH-2020 | ***** | ***** | ***** | ***** | ***** | ***** |  | ***** | 7 |
| Cheng WH-2019 | ***** | ***** | ***** | ***** | ****** | ***** | ***** | ***** | 9 |
| Lee SR-2019 | ***** | ***** | ***** | ***** | ****** | ***** | ***** | ***** | 9 |
| Lee KH -2017 | ***** | ***** | ***** | ***** | ****** | ***** | ***** | ***** | 9 |
| Ikeda T-2019 | ***** | ***** | ***** | ***** | ****** | ***** |  | ***** | 8 |
| Murata N-2019 | ***** | ***** | ***** | ***** | ****** | ***** | ***** | ***** | 9 |
| Arbel R-2019 | ***** | ***** | ***** | ***** | ****** | ***** | ***** | ***** | 9 |
| Briasoulis A-2020 | ***** | ***** | ***** | ***** | ****** | ***** | ***** | ***** | 9 |
| Yao X-2017 | ***** | ***** | ***** | ***** |  | ***** |  | ***** | 6 |
| Steinberg BA-2016 | ***** | ***** | ***** | ***** | ****** | ***** |  | ***** | 8 |
| Camm AJ-2020 | ***** | ***** | ***** | ***** | ****** | ***** |  | ***** | 8 |


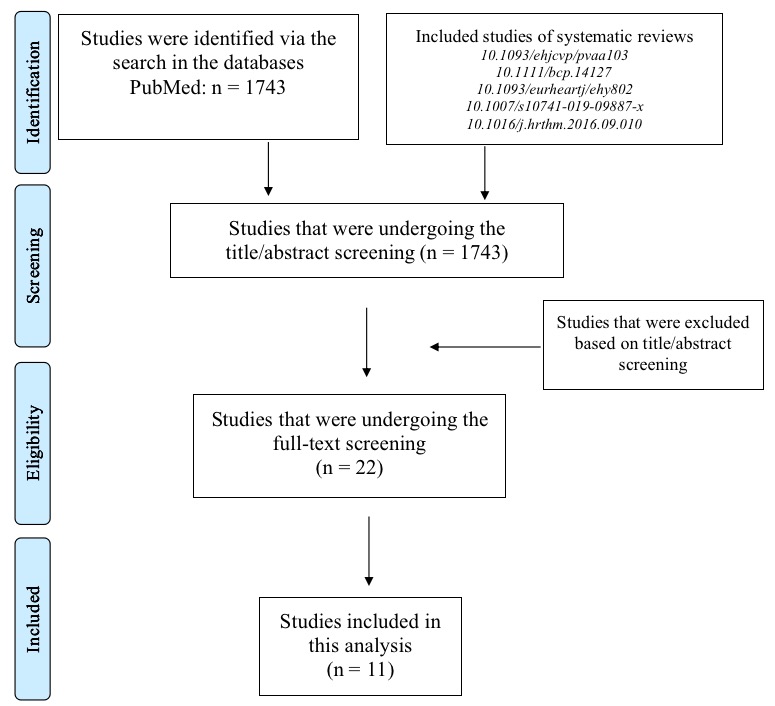


Supplementary Figure 1. The process of electronic retrievals in our meta-analysis


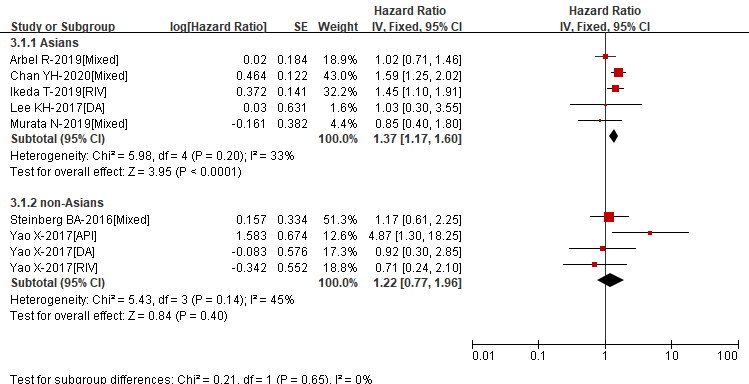


Supplementary Figure 2. Comparing the outcome of stroke or systemic embolism (Asians vs. non-Asians) between non-recommended high dosing and recommended dosing of DOACs


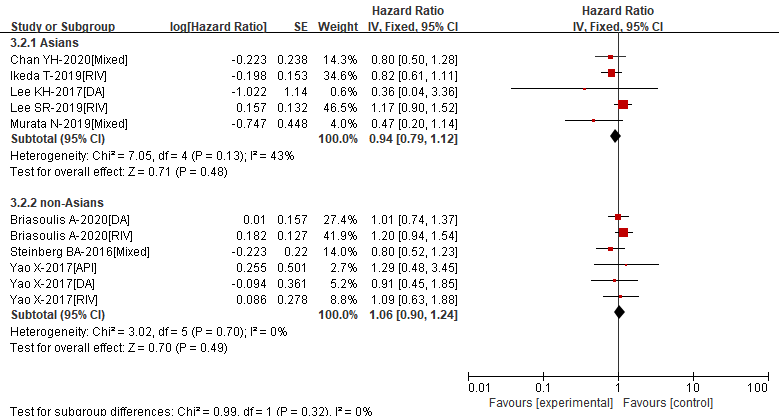


Supplementary Figure 3. Comparing the outcome of stroke or systemic embolism (Asians vs. non-Asians) between non-recommended high dosing and recommended dosing of DOACs


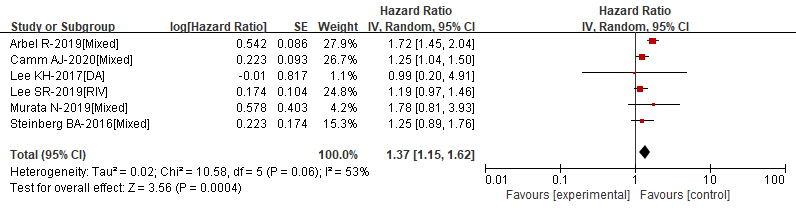


Supplementary Figure 4. Comparing the outcome of all-cause death between non-recommended low dosing and recommended dosing of DOACs in patients with


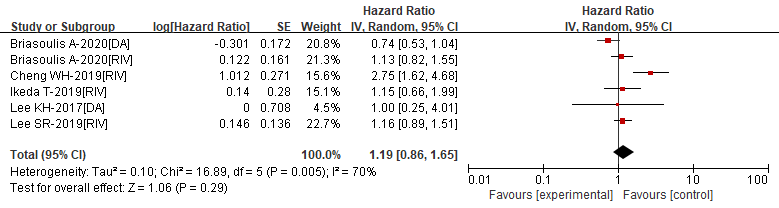


Supplementary Figure 5. Comparing the outcome of ischemic stroke between non-recommended low dosing and recommended dosing of DOACs in patients with AF


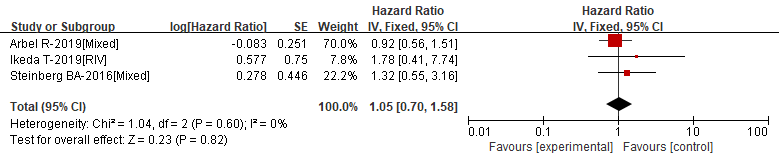


Supplementary Figure 6. Comparing the outcome of myocardial infarction between non-recommended low dosing and recommended dosing of DOACs in patients with AF


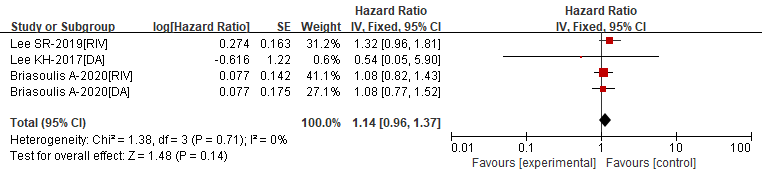


Supplementary Figure 7. Comparing the outcome of gastrointestinal bleeding between non-recommended low dosing and recommended dosing of DOACs in patients with AF


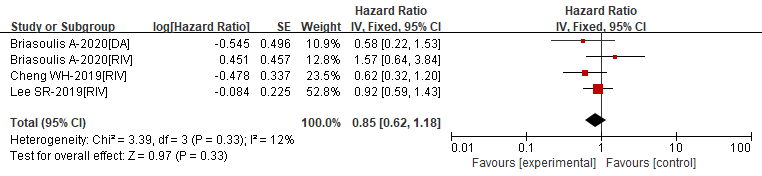


Supplementary Figure 8. Comparing the outcome of intracranial bleeding between non-recommended low dosing and recommended dosing of DOACs in patients with AF


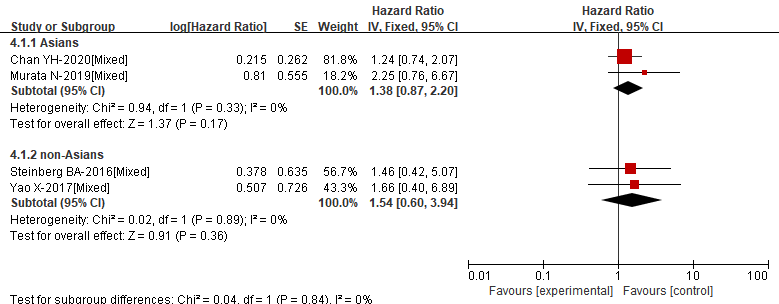


Supplementary Figure 9. Comparing the outcome of stroke or systemic embolism (Asians vs. non-Asians) between non-recommended high dosing and recommended dosing of DOACs


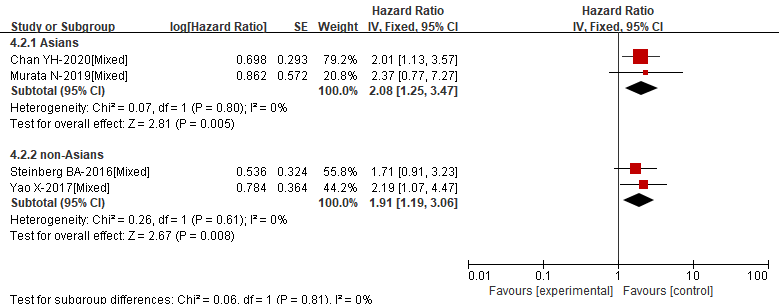


Supplementary Figure 10. Comparing the outcome of major bleeding (Asians vs. non-Asians) between non-recommended high dosing and recommended dosing of DOACs


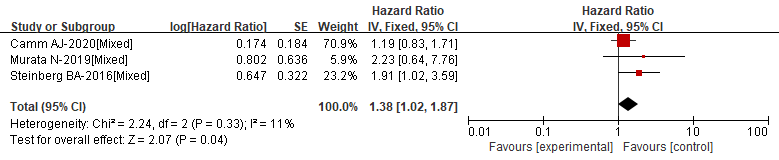


Supplementary Figure 11. Comparing the outcome of all-cause death between non-recommended high dosing and recommended dosing of DOACs in patients with AF


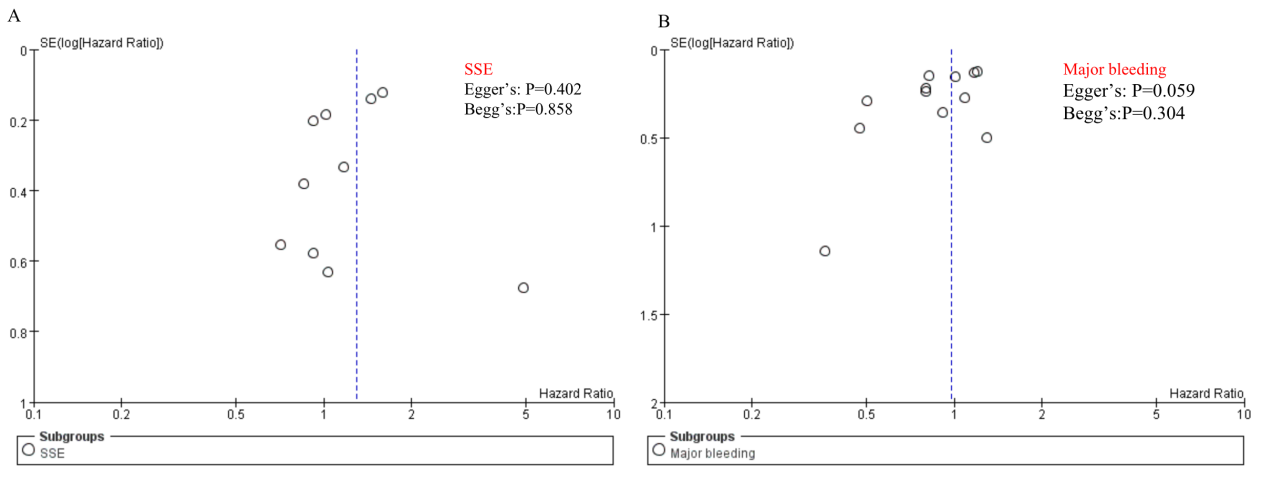


Supplementary Figure 12. Publication bias assessment of the primary outcomes including SSE and major bleeding (non-recommended low dosing versus recommended dosing)

SSE=stroke or systemic embolism; SE=standard error


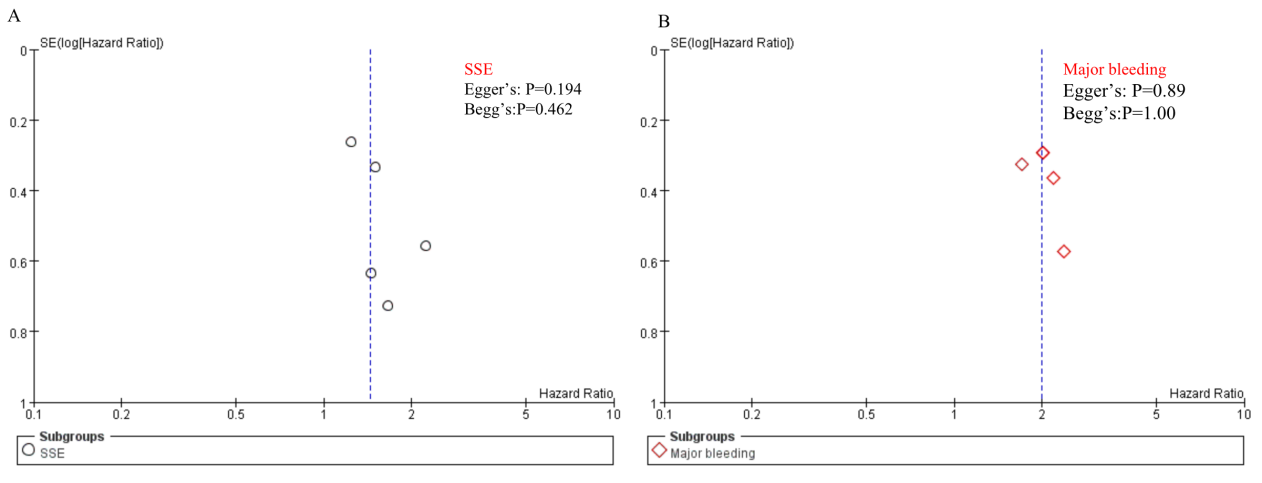


Supplementary Figure 13. Publication bias assessment of the primary outcomes including SSE and major bleeding (non-recommended high dosing versus recommended dosing)

SSE=stroke or systemic embolism; SE=standard error
